# Alda-1 Prevents Pulmonary Epithelial Barrier Dysfunction following Severe Hemorrhagic Shock through Clearance of Reactive Aldehydes

**DOI:** 10.1155/2019/2476252

**Published:** 2019-08-04

**Authors:** Tianfeng Hua, Min Yang, Yangyang Zhou, Limin Chen, Huimei Wu, Rongyu Liu

**Affiliations:** ^1^Department of Geriatric Respiratory and Critical Care, Anhui Geriatric Institute, The First Affiliated Hospital of Anhui Medical University, Hefei, Anhui, China; ^2^The Laboratory of Cardiopulmonary Resuscitation and Critical Care Medicine, The Second Affiliated Hospital of Anhui Medical University, Hefei, China; ^3^Intensive Care Unit, The Second Affiliated Hospital of Anhui Medical University, Hefei, China

## Abstract

Severe hemorrhagic shock and resuscitation (HS/R) can lead to lung injury, resulting in respiratory insufficiency. We investigated whether treatment with Alda-1, an ALDH2 activator, decreased lung injury induced by severe HS/R in a rat model. Male Sprague-Dawley rats were randomized into three groups, hemorrhagic shock + placebo, hemorrhagic shock + Alda-1, and sham. All animals were heparinized, and then 50% of the total calculated blood volume was collected over 60 minutes. After 40 minutes of hemorrhagic shock, animals were reinfused with the shed blood over 40 minutes and then observed for an additional 2 hours. Concentrations of 4-HNE, TNF-*α*, IL-6, and ALDH2 activity were detected; lung injury and lung wet-to-dry weight ratios were assessed. Expression of occludin and ZO-1 proteins in lung tissues was also determined. At 2 hours after resuscitation, lung injury was significantly reduced and the wet-to-dry weight ratio was notably decreased in the Alda-1 group compared with placebo (P<0.05). Alda-1 treatment also significantly increased the activity of ALDH2 and decreased the levels of toxic 4-HNE (P<0.05). In the Alda-1 group, IL-6 and TNF-*α* were dramatically decreased compared with placebo-treated animals (P<0.05). Expression of occludin and ZO-1 proteins was significantly decreased in the placebo group compared with the Alda-1 group (P<0.05). Thus, in a rat model of severe HS/R, treatment with Alda-1 increased the activity of ALDH2, significantly accelerated the clearance of reactive aldehydes, and concomitantly alleviated lung injury through improvement of pulmonary epithelial barrier integrity resulting in decreased alveolar epithelial tissue permeability, lung edema, and diffuse infiltration of inflammatory cells.

## 1. Introduction

Hemorrhage accounts for up to approximately 40% of trauma-related deaths. Besides its role in early mortality, it is known that severe hemorrhagic shock and resuscitation (HS/R) can result in oxidative stress, which contributes to the development of organ injury, especially in the lungs [1]. Lung injury is the most common cause of respiratory insufficiency after severe hemorrhagic shock and the ensuing resuscitation, which induces infiltration of neutrophils to the lung tissue followed by the release of oxygen radicals and proteases, causing impairment of the surrounding parenchyma cells [2–4] and noncardiogenic pulmonary edema [5]. During severe HS/R, excessive reactive oxygen species (ROS) directly contribute to the initial injury and inflammation [6]. Furthermore, ROS-induced oxidative stress injury can be amplified and propagated by toxic aldehydes produced by ROS-triggered peroxidation of unsaturated lipids [7]. Toxic aldehydes, such as 4-hydroxynonenal (4-HNE), are more stable than ROS, further exacerbating pulmonary injury and enhancing the intense inflammatory response. Therefore, detoxification of reactive aldehydes is an endogenous protective mechanism against cell damage resulting from ischemia-reperfusion injury (IRI).

Many studies have shown that aldehyde dehydrogenase 2 (ALDH2) can break down toxic aldehydes into nontoxic substances. Alda-1, the ALDH2 agonist, significantly accelerates the clearance of reactive aldehydes through elevating ALDH-2 activity [8–10]. Many studies have demonstrated that Alda-1 significantly reduces myocardial, cerebral, and liver injury [9, 11, 12]. Alda-1 also has been demonstrated to alleviate lung ischemia-reperfusion injury [13]. However, the effect of Alda-1 on HS/R-induced lung tissue damage is still unknown.

Several recent studies have proposed that injury to the alveolar epithelial barrier increases alveolar permeability, inducing pulmonary edema and subsequent pathological changes. The expression of tight junctions was downregulated in the pathogenesis of acute lung injury and hyperoxia-induced destruction of the pulmonary epithelial barrier [14, 15]. And occludin and zonula occludens 1 [ZO-1] played important functions in maintaining the integrity and barrier function of the tight junctions. Therefore, we hypothesized that increased clearance of reactive toxic aldehydes may decrease injury to the lung tight junctions (occludin and ZO-1), maintain the integrity of the pulmonary epithelial barrier, and therefore decrease alveolar epithelial tissue permeability, lung edema, and diffuse infiltration of inflammatory cells. Agents aiming at decreasing toxic aldehydes to protect tight junctions in the alveolar epithelial barrier may be a novel target for prevention and treatment of lung injury induced by severe HS/R. In the present study, we investigated the effect of Alda-1 on lung injury induced by severe HS/R and the potential mechanisms involved.

## 2. Materials and Methods

### 2.1. Experimental Animals

Twenty healthy male Sprague-Dawley rats, weighing between 350 and 400g, were used in this study. All animals were anesthetized by an intraperitoneal injection of pentobarbital (45mg/kg) and additional doses of pentobarbital (10mg/kg) were required to maintain anesthesia. The trachea of all animals was intubated with the same 14-G cannula (Abbocath-T; Abbott Hospital Products Division, North Chicago, IL) which was mounted on a blunt 45° angled needle. Spontaneous breathing of room air was maintained in all animals and a conventional lead II electrocardiogram was continuously monitored.

Polyethylene catheters (PE-50; Becton Dickinson, Franklin Lakes, NJ) were advanced into the right carotid artery, right femoral artery, and right femoral vein for blood withdrawal, aortic pressure measurement, arterial blood sampling, and blood reinfusion. All catheters were flushed intermittently with saline containing 2.5 IU/mL of crystalline bovine heparin. A thermocouple microprobe (9030-12-D-34; Columbus Instruments, Columbus, OH) was inserted into the inferior cava vena from the left femoral vein for measurement of blood temperature, which was maintained at 37°C ± 0.2°C with infrared surface heating lamps.

All experimental animals received humane care in compliance with the Guide for the Care and Use of Laboratory Animals. This protocol was approved by the Institutional Animal Care and Use Committee of the First Affiliated Hospital of Anhui Medical University.

### 2.2. Experimental Procedures

Following the above preparation, animals were randomized into three groups: (1) hemorrhagic shock + placebo (n=8); (2) hemorrhagic shock + Alda-1 (n=8); (3) sham (n=4). All animals were heparinized with 100 U/kg bovine heparin. The blood volume of each animal was calculated (estimated blood volume [EBV]=6.12/100g body weight), then an estimated 50% of the total blood volume was withdrawn over a period of 60 min. Alda-1 (10 mg/kg) was administrated intraperitoneally (i.p.) at the onset of shock. For the placebo group, the same total volume of placebo (50% polyethylene glycol and 50% dimethyl sulfoxide) [16] was administered. Animals in the sham group underwent laparotomy but without induction of hemorrhagic shock. Blood from the right carotid artery was let into a sterile 20 ml syringe for storage, using a dual syringe pump (Longer Pump LSP01-1A, Longer Corporation, China). Forty minutes after the completion of bleeding, the animals were reinfused with the shed blood over a period of 40 min. The animals were then monitored for an additional 2 h. Electrocardiography, aortic pressures, and temperature were recorded at 5 min intervals during the hemorrhage and resuscitation procedures and every 15 min thereafter. Blood gases and blood samples were collected at baseline, at the end of shock but before reinfusion, and at 1 and 2 h after the completion of infusion. All animals were euthanized by intravenous injection of pentobarbital (150 mg/kg) at 2 h postresuscitation. Routine necropsy was performed for gross documentation of injuries caused by surgical intervention.

### 2.3. Physiological Measurements

Electrocardiogram and aortic pressure values were continuously recorded on a PC-based data-acquisition system supported by WINDAQ software (DATAQ, Akron, OH). Aortic blood pH, pCO_2_, pO_2_, hemoglobin, and lactate concentrations were measured in 0.2ml blood with a Stat Profile pHOx Plus analyzer (Model RADIOMETER ABL80FLEX; Radiometer Medical ApS, Bronshoj, Denmark). Blood samples were collected from arterial blood and centrifuged at 3000×g for 10 mins to obtain serum. Serum and lung samples were stored at −80°C for future analysis. Additionally, lung tissue samples were stored in 4% paraformaldehyde for histopathology and immunochemistry.

### 2.4. Lung Wet-to-Dry Weight Ratio

The lung wet-to-dry weight ratio was assessed as previously described [17]. Briefly, after euthanasia, the inferior lobe of the left lung was harvested immediately, then weighed and heated to 65°C in a constant temperature oven for 72 hrs. The wet-to-dry weight ratio of lungs was calculated at the end of the experiment.

### 2.5. Lung Histopathologic Analysis

Lung tissue from randomly selected rats within each group was sampled and fixed immediately after the end of each experiment in 4% paraformaldehyde and then embedded in paraffin. Tissue sections were stained with hematoxylin and eosin (H&E) and evaluated under light microscopy. The lung histological injury was scored by evaluating the intra-alveolar congestion, intra-alveolar hemorrhage, intra-alveolar, and interstitial infiltration of leukocytes and the thickness of the alveolar wall/hyaline membrane [18]. Twenty high-magnification fields were randomly selected at × 400 magnification within each field. Histopathological evaluation was analyzed by three independent observers who were blinded to the experiment.

### 2.6. Measurement of 4-HNE Concentration and ALDH2 Activity

4-HNE concentration and ALDH2 activity were measured using 4-HNE enzyme-linked immunosorbent assay (ELISA) and ALDH2 activity assay kits, respectively (Wuhan CUSABIO Biotech Industry Co. Ltd., China and Sigma-Aldrich, St. Louis, MO, USA) following the manufacturer's protocols. ALDH2 activity was determined by a coupled enzyme assay in which acetaldehyde is oxidized, generating a colorimetric product proportional to the ALDH2 activity present. The results were read at 450 nm on a plate reader (TECAN GENios, Austria).

### 2.7. Cytokine Determination in Lung Tissues

One hundred milligrams of frozen lung tissue were rinsed and homogenized in 1ml of 1×PBS and stored at −20°C overnight. After two freeze-thaw cycles were performed to disrupt the cell membranes, the homogenates were centrifuged at 5000×g for 5 min and the supernatant was then used for cytokine analysis. The cytokines in lung tissue were measured using ELISA kits specific for rat tumor necrosis factor (TNF)-*α* and interleukin-6 (IL-6) (Wuhan CUSABIO Biotech Industry Co. Ltd., China) following the manufacturer's instructions.

### 2.8. Occludin and ZO-1 Protein Expression in Lung Tissues

We detected occludin and ZO-1 protein expression by immunohistochemistry. The tissue sections were incubated with antioccludin and anti-ZO-1 antibody (both 1:400, Invitrogen, San Francisco, CA, USA) at 4°C overnight and then with a biotinylated secondary antibody. Freshly prepared 3,3′-diaminobenzidine (DAB) was used to visualize the antigen–antibody reaction. Brown granules represented positive cells. Ten high-magnification fields were randomly selected at ×400 magnification within each field. Brown granules were analyzed by three independent observers who were blinded to the experiment.

### 2.9. Statistical Analysis

Data are presented as mean ± standard deviation if they were normally distributed. Comparisons between time-based measurements within each group were performed by repeated-measurement analysis of variance. If there was a significant difference in the overall comparison of groups, comparisons among multiple groups were made using a one-way analysis of variance (ANOVA) followed by Scheffe post hoc test. P values of <0.05 were considered statistically significant.

## 3. Results

Twenty-three rats were used in this study; however, three of them were excluded for instrumentation or technical failure before randomization. Twenty rats were thus investigated for completion of the experiment. There were no significant differences in baseline characteristics of body weight, body temperature, hemodynamic parameters, blood gas, or arterial blood lactate between the three treatment groups (Table 1).

**Figure 1:**
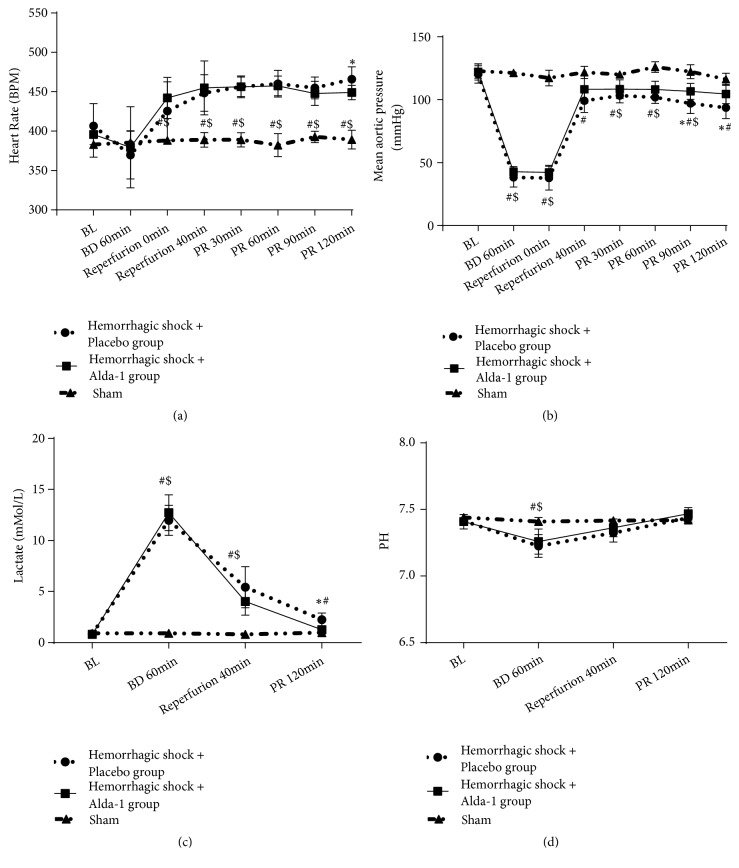
Changes in heart rate (a), mean arterial pressure (b), lactate (c), and pH (d) during hemorrhagic shock and resuscitation in the three treatment groups, hemorrhagic shock + placebo, hemorrhagic shock + Alda-1 treatment, and sham. BL: baseline; BD: blood withdrawn; PR: postresuscitation. *∗*P<0.05, hemorrhagic shock + placebo versus hemorrhagic shock + Alda-1; #P<0.05, hemorrhagic shock + placebo versus sham; $P<0.05, hemorrhagic shock + Alda-1 versus sham.

During the hemorrhage and shock phases, the mean arterial pressure (MAP) was significantly decreased in both HS/R groups when compared with the sham group (all P<0.05). During the resuscitation phase, MAP was then quickly restored to near-baseline levels. In addition, we found that MAP was significantly better in the Alda-1 group compared with placebo (all P<0.05; Figure 1).

### 3.1. Lung Wet-to-Dry Weight Ratio

**Figure 2:**
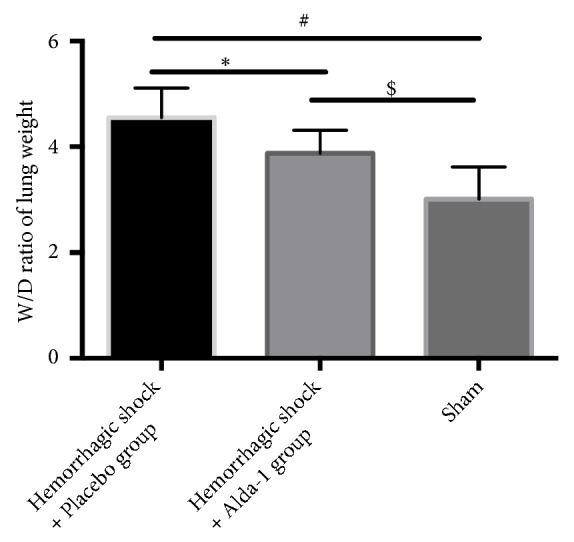
Wet-to-dry (W/D) weight ratio in lung tissue in treated animals. *∗*P<0.05, hemorrhagic shock + placebo versus hemorrhagic shock + Alda-1; #P<0.05, hemorrhagic shock + placebo versus sham; $P<0.05, hemorrhagic shock + Alda-1 versus sham.

The lung wet-to-dry weight ratios were significantly increased at 2hrs after postresuscitation in both HS/R groups when compared with the sham group (all P<0.05). However, the wet-to-dry ratio was significantly lower in the Alda-1 group compared with the placebo group (P<0.05; Figure 2).

### 3.2. Alda-1 Protected against Lung Tissue Morphological Injuries

**Figure 3:**
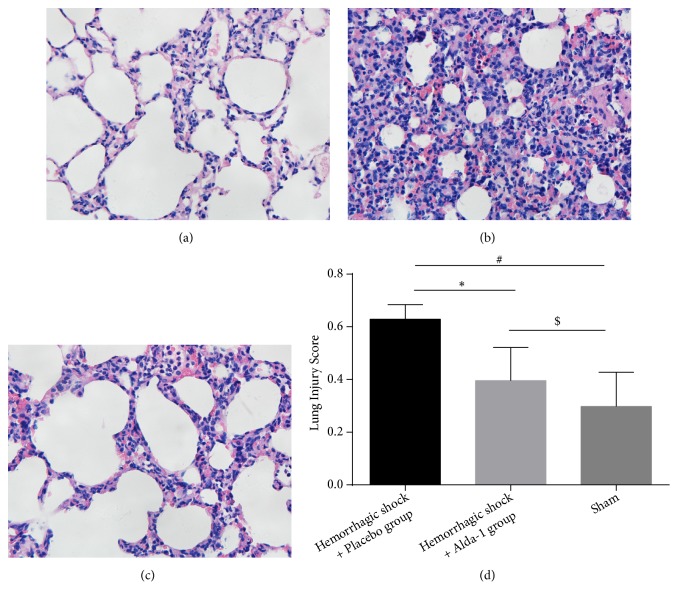
Lung injury in (a) sham, (b) hemorrhagic shock + placebo, and (c) hemorrhagic shock + Alda-1-treated rats (magnification, × 400). Lung injury was defined as intra-alveolar congestion, intra-alveolar hemorrhage, intra-alveolar and interstitial infiltration of leukocyte infiltration and thickening of the alveolar wall/hyaline membrane. Lung injury scores of sham, hemorrhagic shock + placebo, and hemorrhagic shock + Alda-1 treated rats are presented in (d). *∗*P<0.05, hemorrhagic shock + placebo versus hemorrhagic shock + Alda-1; #P<0.05, hemorrhagic shock + placebo versus sham; $P<0.05, hemorrhagic shock + Alda-1 versus sham.

To determine the effects of Alda-1 on lung tissue after severe HS/R, we conducted lung histological analysis by H&E staining (magnification, × 400). The lung injury scores were determined by assessing alveolar congestion, exudates, hemorrhage, and infiltration of neutrophils. HS/R induced this lung injury, but administration of Alda-1 ameliorated these indicators of damage, instead demonstrating better aerated alveoli, fewer infiltrating neutrophils and reduced intra-alveolar congestion, exudates, and hemorrhage. The lung injury scores were significantly decreased by 38.7% in the Alda-1-treated animals compared with the hemorrhagic shock + placebo group, but increased compared with sham-treated rats (P<0.05; Figure 3).

### 3.3. Alda-1 Increased ALDH2 Activity and Decreased 4-HNE Levels

**Figure 4:**
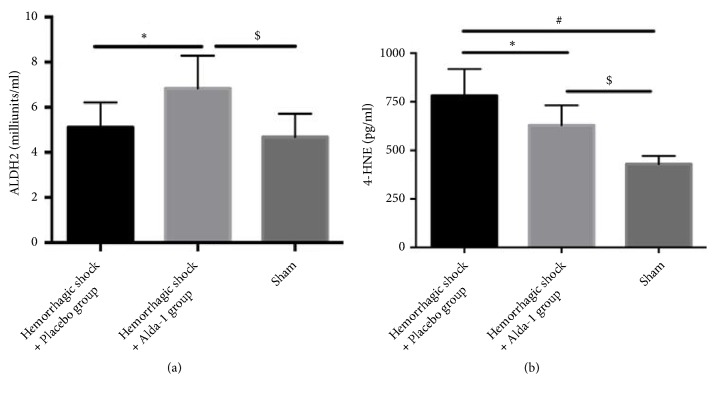
ALDH2 activity (a) and 4-HNE levels (b) in lung tissues from hemorrhagic shock + placebo, hemorrhagic shock + Alda-1, and sham groups. *∗*P<0.05, hemorrhagic shock + placebo versus hemorrhagic shock + Alda-1; #P<0.05, hemorrhagic shock + placebo versus sham; $P<0.05, hemorrhagic shock + Alda-1 versus sham group.

The data demonstrated that HS/R did not decrease the activity of ALDH2. However, Alda-1 significantly increased the activity of ALDH2. Furthermore, severe HS/R elevated levels of the toxic aldehyde 4-HNE in both shock groups compared with the sham group, but the 4-HNE concentration was dramatically lower in the Alda-1 group compared with placebo-treated rats (P<0.05; Figure 4).

### 3.4. Alda-1 Decreased IL-6 and TNF-*α* Levels

**Figure 5:**
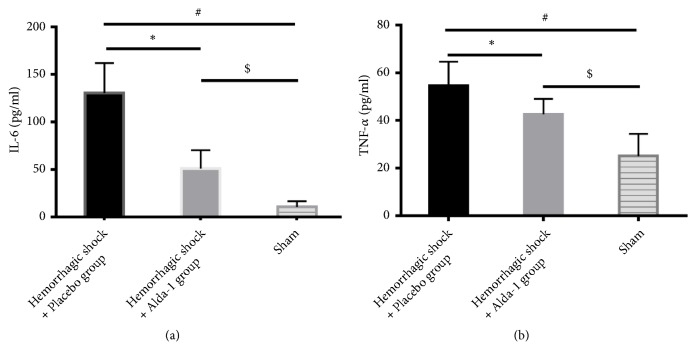
IL-6 (a) and TNF-*α* (b) levels in lung tissues from hemorrhagic shock + placebo, hemorrhagic shock + Alda-1, and sham groups. *∗*P<0.05, hemorrhagic shock + placebo versus hemorrhagic shock + Alda-1; #P<0.05, hemorrhagic shock + placebo versus sham; $P<0.05, hemorrhagic shock + Alda-1 versus sham.

The results showed that severe HS/R elevated IL-6 and TNF-*α* levels in both shock groups. However, compared with the placebo group, treatment with Alda-1 significantly reduced the IL-6 and TNF-*α* concentrations (P<0.05; Figure 5).

### 3.5. Alda-1 Improved Expression of Occludin and ZO-1 Proteins

**Figure 6:**
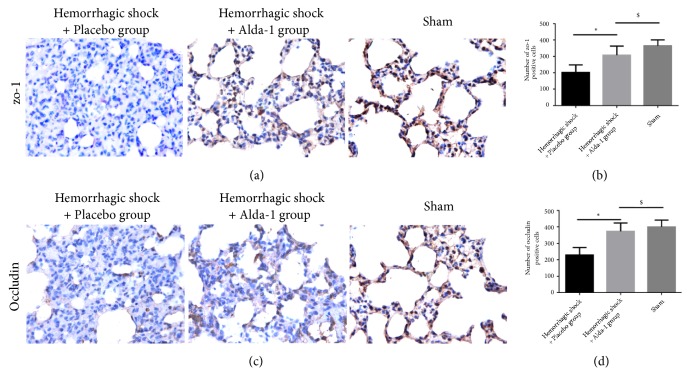
Expression of ZO-1 (a) and occluding (c) proteins in hemorrhagic shock + placebo, hemorrhagic shock + Alda-1, and sham-treated rats with immunohistochemistry (magnification, × 400). ZO-1 and occluding were quantified by brown granules which represent positive cells (b, d). *∗*P<0.05, hemorrhagic shock + placebo versus hemorrhagic shock + Alda-1; #P<0.05, hemorrhagic shock + placebo versus sham; $P<0.05, hemorrhagic shock + Alda-1 versus sham.

In order to explain the mechanism of the observed lung-protective effect by Alda-1 in this severe HS/R in vivo model, we investigated the expression of occludin and Zo-1 proteins, both of which are involved in maintenance of tight junctions in the lung, by immunochemistry. We found that severe HS/R significantly decreased the expression of both occludin and ZO-1 and destroyed the integrity of the pulmonary epithelial barrier. Treatment with Alda-1 significantly increased the expression of these proteins and partially maintained the integrity of pulmonary epithelial barrier (P<0.05; Figure 6).

## 4. Discussion

In the present study, we have demonstrated that administration of Alda-1 significantly decreases lung injury and alleviates lung edema following severe HS/R. It is likely that the potential mechanism involves Alda-1-mediated induction of ALDH2 activity [9, 10] which in turn decreases the accumulation of reactive aldehydes and inflammatory factors, thus significantly attenuating damage to tight junction proteins, ultimately maintaining the integrity of the pulmonary epithelial barrier.

Respiratory insufficiency is the most common and inevitable complication following severe HS/R, during which the imbalance between lung oxygen supply and consumption can lead to excessive oxidative stress. Generation of ROS occurs in most pulmonary cells, including endothelial and alveolar epithelial cells. Furthermore, infiltration of various inflammatory cells, which also produce ROS, further increases the ROS levels in the lung tissue. Ultimately, this not only causes direct damage to local cells and tissues, but also results in the production of toxic aldehydes, which are more stable than ROS and thus further propagate and amplify the oxidative stress injury resulting from ischemia-reperfusion [11, 19]. Previous studies have proven that 4-HNE levels were positively correlated with IRI [11]. Likewise, we found that 4-HNE was significantly increased during severe HS/R, with concomitant increases in histological lung injury score and lung wet-to-dry weight ratio in both groups suffering from severe HS/R when compared with the sham group.

ALDH2, a tetrameric enzyme, detoxificated the toxic aldehydes generated from oxidative stress such as 4-HNE [20, 21]. And the levels of reactive acetaldehydes were correlated with outcomes after cardiac I/R injury. Another study further demonstrated that heart protection effect induced by acetaldehydes was through translocating €PKC to mitochondria and activating ALDH2 [22]. ALDH2 exists in many organs including the lung [23, 24]. Here we found that HS/R dramatically induced 4-HNE production; however, the activity of ALDH2 was not significantly decreased within lung tissues during severe H/R. The most likely explanation for the resulting lung damage would be that production of reactive toxic aldehydes such as 4-HNE far surpasses the clearance capacity of ALDH2 during HS/R. Accelerating the removal of aldehydes has been proven to protect the organ from IRI in many animal models. If this also occurs in the lung suffering from HS/R, then it could explain the lung-protective effects of Alda-1 observed in our model. In fact, Alda-1 treatment significantly enhanced the activity of ALDH2, successfully accelerating the clearance of toxic aldehydes, consistent with the results of the wet-to-dry weight ratio, abnormal secretion of inflammatory cytokines and neutrophil infiltration observed in lung tissue [25, 26], which supported our hypothesis. To the best of our knowledge, this is the first study to show that activating ALDH2 by administration of Alda-1 could successfully attenuate pulmonary injury induced by severe HS/R.

Additionally, we further explored the potential mechanism of the observed protective effect of Alda-1. The pathogenesis of pulmonary injury that occurs in severe HS/R is characterized by increased pulmonary permeability, noncardiogenic pulmonary edema, diffuse infiltration of various inflammatory cells, and pulmonary atelectasis [27]. Previous studies have demonstrated that damage to the alveolar epithelial barrier would lead to such pathogenesis [14, 28, 29]. The alveolar epithelial barrier is composed of a monolayer of alveolar epithelial cells and tight junctions between the alveoli and fluid-filled tissue to maintain normal gas exchange. The paracellular tight junctions are responsible for intercellular sealing and regulation of epithelial permeability [15]. Tight junctions include cytoplasmic and transmembrane proteins, together with alveolar epithelial cells to form the alveolar epithelial barrier. ZO-1 and occludin are recognized as two characteristic proteins within tight junctions. Occludin is a transmembrane protein important for stability and barrier function in the tight junction [30, 31]. ZO-1 is an intracellular protein that connects occludin to cytoskeletal proteins and affects paracellular permeability [32]. Disruption of the alveolar epithelial barrier integrity and subsequent increased alveolar epithelial permeability results in alveolar flooding, pulmonary edema, and pathological alterations [33]. Previous studies have demonstrated that 4-HNE triggers apoptosis of human pulmonary alveolar cells in vitro and increases pulmonary alveolar capillary barrier permeability [13]. Acrolein has been proven to increase bronchial epithelial barrier permeability [34] and to decrease expression of the tight junction protein Claudin 5 in an endothelial cell line [35]. However, permeability of the alveolar epithelial barrier is lower than that of the vascular endothelial barrier and plays a decisive role in the development of lung edema [15]. Thus, it is essential to investigate the effect of Alda-1 on alveolar epithelial barrier function. In fact, we observed that the expression of occludin and ZO-1 proteins was significantly decreased during severe HS/R and administration of Alda-1 successfully increased expression of these proteins, thus partly alleviating the damage to the alveolar epithelium integrity and protecting alveolar epithelial barrier permeability. This resulted in decreased lung edema and infiltration of neutrophils and ultimately alleviated lung injury.

As demonstrated in our research, severe HS/R significantly elevated the levels of IL-6 and TNF-*α* [36]. TNF-*α* interferes with the transcription and intracellular localization of tight junction proteins [37]. Following the removal of 4-HNE, expression of IL-6 and TNF-*α* was significantly decreased. Therefore, we have reason to speculate that 4-HNE also interferes with the expression of tight junction proteins through induction of inflammatory mediators during severe HS/R-induced pulmonary injury.

There are several limitations in our research. Firstly, only healthy male rats without disease were used, which does not reflect the clinical situation of human patients. Secondly, toxicity testing is required before the compound can be progressed for clinical use. Thirdly, the survival rate of animals and larger randomized studies must be investigated.

## 5. Conclusions

In summary, our study demonstrated that treatment with Alda-1 significantly protected the lung against severe HS/R-induced injury. The potential mechanism could be through activation of ALDH2 to detoxify 4-HNE, thus alleviating damage to tight junctions in the alveolar epithelium and therefore protecting the alveolar epithelial barrier, ultimately leading to reduced lung edema and injury.

## Figures and Tables

**Table 1:** Baseline characteristics of experimental animals.

Measurement	Sham	Hemorrhagic shock + Placebo group	Hemorrhagic shock + Alda-1 group
Body weight (g)	362.50 ± 6.45	365.00 ± 9.26	365.00 ± 10.00
Heart rate (beats·min-1)	383.00 ± 16.46	406.50 ± 28.46	395.75 ± 12.88
MAP (mmHg)	122.75 ± 5.68	120.12 ± 6.87	122.12 ± 6.49
Lactate (mmol/L)	0.90 ± 0.18	0.84 ± 0.38	0.80 ± 0.31
PH	7.44 ± 0.02	7.41 ± 0.02	7.41 ± 0.05

MAP, mean aortic pressure. Values are presented as mean ± SD.

## Data Availability

The data used to support the findings of this study are included within the article.
